# The Mechanism of Leptin on Inhibiting Fibrosis and Promoting Browning of White Fat by Reducing ITGA5 in Mice

**DOI:** 10.3390/ijms222212353

**Published:** 2021-11-16

**Authors:** Yuexia Liu, Yizhou Li, Juntong Liang, Zhuwen Sun, Qiong Wu, Yongnian Liu, Chao Sun

**Affiliations:** 1 College of Animal Science and Technology, Northwest A&F University, Xianyang 712100, China; nwafuliuyuexia@126.com (Y.L.); liyizhou@nwafu.edu.cn (Y.L.); ljtsci@126.com (J.L.); szw13043277456@163.com (Z.S.); 13997126828@163.com (Q.W.); 2 Medical College, Qinghai University, Xining 810000, China

**Keywords:** adipocyte, leptin, ITGA5, fibrosis, white fat browning

## Abstract

Leptin is a small molecule protein secreted by adipocytes, which can promote white fat browning through activating the hypothalamic nervous system and inhibiting downstream signaling pathways. Moreover, white fat browning has been proven to alleviate fat tissue fibrosis. This study explores the mechanism of leptin in regulating adipose tissue fibrosis and white fat browning. After treating mice with leptin, we screened out the recombinant integrin alpha 5 (ITGA5) through proteomics sequencing, which may play a role in adipose tissue fibrosis. Through real-time quantitative PCR (qPCR), western blotting (WB), hematoxylin-eosin (HE) staining, Masson’s trichrome, immunofluorescence, immunohistochemistry, etc., the results showed that after leptin treated adipocytes, the expression of fibrosis-related genes and ITGA5 was significantly down-regulated in adipocytes. We constructed fibrosis model through transforming growth factor-β (TGF-β) and a high-fat diet (HFD), and treated with ITGA5 overexpression vector and interference fragments. The results indicated the expression of fibrosis-related genes were significantly down-regulated after interfering with ITGA5. After treating adipocytes with wortmannin, fibrosis-related gene expression was inhibited after overexpression of ITGA5. Moreover, after injecting mice with leptin, we also found that leptin significantly up-regulated the expression of adipose tissue browning-related genes. Overall, our research shows that leptin can inhibit the activation of phosphatidylinositol 3 kinase (PI3K)-protein kinase B (AKT) signaling pathway by reducing the expression of ITGA5, which could alleviate adipose tissue fibrosis, and further promote white fat browning. Our research provides a theoretical basis for further research on the effect of leptin in fibrosis-related adipose tissue metabolism.

## 1. Introduction

Adipose tissue has an important energy storage function in the body. According to the source, shape, and function, adipocytes can be divided into three types: white adipocytes, brown adipocytes, and beige adipocytes [1]. White adipocytes play an important role in energy storage, and studies have found adiponectin and leptin are two important cytokines secreted by white adipocytes, which play crucial roles in a variety of life processes [2]. Brown adipocytes mainly rely on the abundant uncoupling protein 1 (UCP1) in the mitochondria to leak the proton pump on the oxidative respiratory chain in the mitochondria, thereby converting energy into heat to deal with the body’s cold stimulation [3]. Beige adipocytes are a type of cell formed by the body’s transformation from white adipocytes to brown adipocytes in a cold environment or other specific conditions. Beige adipocytes also have multiple lipid droplets, and the content of mitochondria and UCP1 is higher than that of white adipocytes. This process is called white fat browning [3,4]. White fat browning is conducive to the consumption of excess lipids stored in white adipose tissue, reducing obesity, and also improving adipocyte tissue inflammation [1,5].

Leptin was discovered and named in mice in 1994. It is mainly secreted form adipose tissue and has been widely studied as a small molecule peptide [6]. The structural feature of leptin is that the four main α helices of A, B, C, and D form a spiral bundle in the form of “rising-falling-falling-falling”, and the inside is fixed by disulfide bonds [7]. After leptin is secreted into blood circulation, it mainly exists as a monomer [8]. Researchers found that leptin affected appetite by acting on the hypothalamus, and it can also stimulate sympathetic nerve activity, promoting the increase of peripheral norepinephrine secretion, thereby activating β3 adrenergic receptors on adipocytes, causing adipocytes to produce a lot of energy and reduce body fat [9,10]. Moreover, leptin can directly bind to the leptin receptor on adipocytes, thereby inhibiting the lipid synthesis of adipocytes [11].

Leptin, as an important adipose factor secreted by adipocytes, plays an important role in the process of white fat browning. Studies have found that leptin can reduce the expression of neuropeptide Y (NPY) by acting on hypothalamic neurons, and the lack of NPY can promote white fat browning [12]. Meanwhile, leptin can stimulate the activation of sympathetic nerves and promote the increase of norepinephrine secretion, thereby activating β3 adrenergic receptors on adipocytes, resulting in white fat browning [13]. Researchers found that leptin also activated the expression of pr domain-containing 16 (PRDM16) through its downstream Janus kinase 2 (JAK2)-signal transducer and activator of transcription (STAT3) signaling pathway, thereby promoting white fat browning [14]. Moreover, it can also promote white fat browning by inhibiting the Hedgehog (Hh) signaling pathway [15].

In addition to lipid metabolism, leptin plays an important role in regulating fibrosis in tissues or organs. At present, studies have found that leptin is important in the fibrosis of multiple peripheral organs [16,17]. Adipose tissue fibrosis is the long-term exposure of adipose tissue in an environment of hypoxia and chronic inflammation, which leads to a state of continuous remodeling of the extracellular matrix (ECM) of the adipose tissue, and ultimately leads to excessive deposition of ECM in the adipose tissue, thereby resulting in adipose tissue metabolism disorder. Moreover, obesity is generally one of the main factors leading to fibrosis of adipose tissue [18,19]. In adipose tissue fibrosis, the extracellular matrix is mainly derived from a variety of cells, such as adipocytes and macrophages. As an important adipocyte factor, leptin has an important effect on lipid metabolism and inflammation of adipose tissue [20]. Leptin can reduce the expression of the collagen VI (COL6) in human adipose tissue and ob/ob mice, which, lacking leptin, are often accompanied by adipose tissue fibrosis; however, adding exogenous leptin to ob/ob mice can significantly alleviate adipose tissue fibrosis [21,22]. In obese mice fed high-fat diets (HFD) and ob/ob mice, adipose tissue will be accompanied by fibrosis. After injection of leptin, it can alleviate the inflammation of adipose tissue and reduce the expression of fibrosis-related genes [23,24].

Adipose tissue fibrosis is closely related to white fat browning. In the process of white fat browning, PRDM16 promotes fatty acid oxidation to increase the secretion of β-hydroxybutyric acid from adipocytes, and promotes the differentiation of pre-adipocytes toward beige adipocytes, inhibits its differentiation toward fibroblasts, thereby alleviating adipose tissue fibrosis [25]. During the browning process, PRDM16 binds to the transcription factor general transcription factor II–I repeat domain-containing protein 1 (GTF2IRD1), thereby inhibiting the expression of adipocyte fibrosis-related genes and alleviating adipose tissue fibrosis [26]. Current research have found that the process of white fat browning can effectively alleviate the fibrosis of adipose tissue [25,26].

Integrin α5 (ITGA5) is an integrin α5 subunit, a member of the integrin family, and is often combined with β1 subunit through non-covalent bonds. ITGA5 not only has the function of connecting the extracellular matrix (ECM) and intracellular skeletal network, but also it is a receptor for fibronectin and fibrinogen, and recognizes the sequence R-G-D in its ligands.. ITGA5 mediates signal communication between ECM and cells, and regulates cell differentiation and migration [27,28]. At the same time, ITGA5 is closely related to the adipogenic differentiation of adipocytes, and overexpression of ITGA5 will promote the differentiation of preadipocytes into mature adipocytes [29]. Studies have found that ITGA5 is also closely related to tissue fibrosis, when the expression of ITGA5 is inhibited, liver fibrosis will be alleviated [30]. Therefore, this study explores whether leptin can affect the adipose tissue fibrosis and white fat browning through ITGA5, and its regulatory mechanism, and provides a theoretical basis for further exploring the mechanism of leptin in regulating adipose tissue fibrosis and white fat browning.

## 2. Results

### 2.1. Leptin Relieves Adipose Tissue Fibrosis in Mice

**Figure 1:**
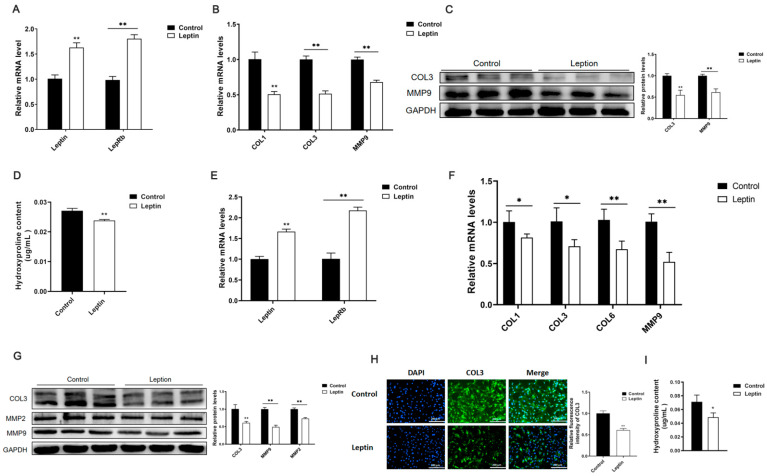
Leptin inhibits adipose tissue fibrosis in mice. When the mice were injected with leptin for 7 days, we extracted total RNA and protein from the inguinal adipose tissue of the mice. (**A**) The relative mRNA expression level of leptin and its receptor (*n* = 4). (**B**) The relative mRNA expression levels of fibrosis-related genes COL1, COL3, and MMP9 (*n* = 4). (**C**) The relative expression levels of fibrosis-related proteins COL3 and MMP9 (*n* = 3). (**D**) The relative expression level of hydroxyproline in adipose tissue (*n* = 4). Furthermore, we respectively treated the cells with 100 ng/mL leptin for 24 and 48 h, and extracted total RNA and protein. (**E**) The relative mRNA expression levels of leptin and its receptor (*n* = 4). (**F**) The relative mRNA expression levels of fibrosis-related genes COL1, COL3, COL6, MMP9 (*n* = 4). (**G**) The relative protein expression levels of fibrosis-related genes COL3, MMP2, MMP9 (*n* = 4). (**H**) Immunofluorescence staining of COL3 in adipocytes, scale bar: 200 μm (*n* = 3). (**I**) The relative expression level of hydroxyproline in adipocytes (*n* = 4). Values are means ± SD, * *p* < 0.05, ** *p* < 0.01 compared with the control group.

To investigate the effect of leptin on adipose tissue fibrosis, we first injected mice with leptin for 7 days, and extracted the total RNA and protein of the mouse inguinal adipose tissue. It was found that leptin treatment promoted the mRNA levels of leptin and leptin receptors in mice (Figure 1A). At the same time, the mRNA levels of collagen I (COL1), collagen III (COL3), and matrix metalloproteinase 9 (MMP9) (Figure 1B), and the protein levels of COL3 and MMP9 (Figure 1C), were suppressed. Further testing found that leptin treatment significantly inhibited the hydroxyproline content in the inguinal fat tissue of the mice (Figure 1D).

Furthermore, we discussed the effect of leptin on adipocyte fibrosis. We added 100 ng/mL leptin to the culture medium when 3T3-L1 cells reached 80%, respectively, treated them for 24 and 48 h, and extracted total RNA and protein. The results showed that after treating cells with leptin, it promoted the mRNA expression level of leptin and its receptors in the cells (Figure 1E), inhibited the mRNA expression levels of fibrosis-related genes COL1, COL3, COL6, and MMP9 (Figure 1F), and inhibited COL3 and protein expression of MMP9 (Figure 1G). Furthermore, cellular immunofluorescence showed that leptin treatment inhibited the expression of COL3 protein in adipocytes (Figure 1H). Afterwards, it was treated with leptin; the content of hydroxyproline in the cells was also significantly reduced (Figure 1I).

Based on the results of these experiments, it was found that leptin could significantly reduce the expression of adipose tissue fibrosis-related genes, which indicates that leptin can alleviate adipose tissue fibrosis.

### 2.2. Leptin Relieves Adipose Tissue Fibrosis by Inhibiting ITGA5

**Figure 2:**
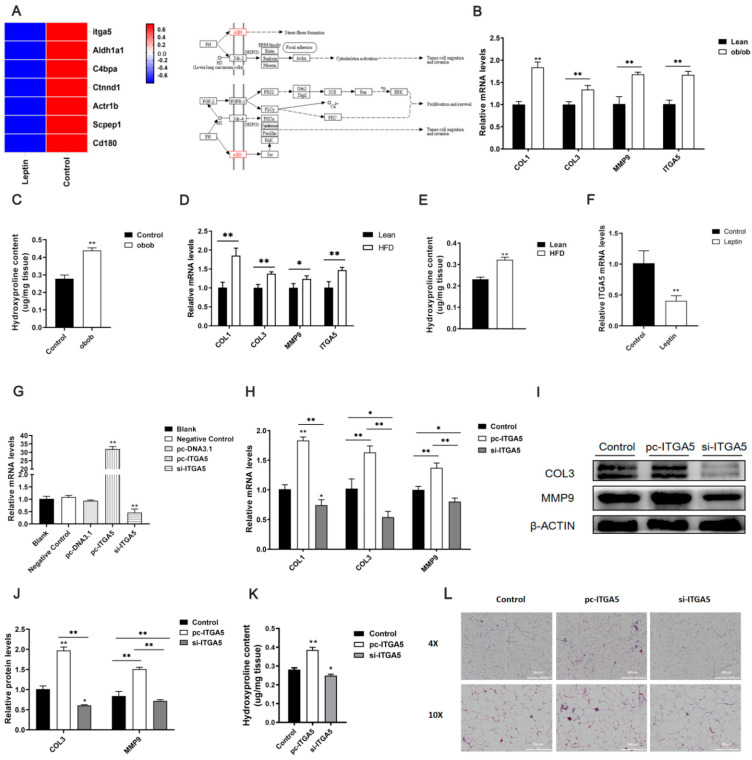
Leptin relieves adipose tissue fibrosis by inhibiting ITGA5. (**A**) Proteomics sequencing analysis (*n* = 4). (**B**) The relative mRNA expression levels of ob/ob mice adipose tissue fibrosis-related genes COL1, COL3, MMP9, and ITGA5 (*n* = 4). (**C**) The relative content of hydroxyproline in adipose tissue of ob/ob mice (*n* = 4). (**D**) The relative mRNA expression levels of adipose tissue fibrosis-related genes COL1, COL3, MMP9, and ITGA5 in HFD mice (*n* = 4). (**E**) The relative content of hydroxyproline in adipose tissue of HFD mice (*n* = 4). (**F**) Afterwards, it is treated with leptin, the relative expression levels of ITGA5 mRNA in mice adipose tissue (*n* = 4). (**G**) After interference and overexpression of ITGA5 in mice, the expression efficiency of ITGA5 in adipose tissue (*n* = 4). (**H**) After interference and overexpression of ITGA5 in mice, relative mRNA expression levels of adipose tissue fibrosis-related genes COL1, COL3, MMP9 (*n* = 4). (**I**,**J**) After interference and overexpression of ITGA5 in mice, the relative mRNA expression levels of adipose tissue fibrosis-related genes COL3, MMP9 (*n* = 4). (**K**) After interference and overexpression of ITGA5 in mice, the relative content of hydroxyproline in adipose tissue (*n* = 4). (**L**) After interference and overexpression of ITGA5 in mice, Masson staining of mouse adipose tissue, scale bar: 300 μm, scale bar: 200 μm (*n* = 4). Values are means ± SD, * *p* < 0.05, ** *p* < 0.01 compared with the control group.

Through the above experiments, we found that leptin could alleviate adipose tissue fibrosis. To further explore the mechanism of leptin alleviating adipocyte fibrosis, we processed adipocyte with leptin for proteomics sequencing. Through GO and KEGG analysis, we screened out the protein integrin α5 (ITGA5), which is closely related to fibrosis, as the target protein (Figure 2A). We found that the mRNA expression levels of fibrosis-related genes COL1, COL3, and MMP9 were significantly increased in HFD and ob/ob mice, and the mRNA level of ITGA5 was also significantly increased (Figure 2B,D). Moreover, the content of hydroxyproline also significantly increased in adipose tissue of ob/ob and HFD mice (Figure 2C,E). At the same time, we detected that the expression of ITGA5 was significantly down-regulated in the adipose tissue of mice treated with leptin (Figure 2F). This indicated that the adipose tissue of obese mice was often accompanied by fibrosis, and ITGA5 may be closely related to obesity and fibrosis. At the same time, we speculated that the function of leptin to relieve fibrosis of adipose tissue might be related to ITGA5.

To further explore the effect of ITGA5 on adipose tissue fibrosis, we first constructed ITGA5 overexpression vector and siRNA fragments, and injected mice. It was found that ITGA5 overexpression and interference efficiency were extremely significant (Figure 2G). Further research found that ITGA5 could promote the mRNA expression levels of adipose tissue fibrosis-related genes COL1, COL3, MMP9, and the protein expression levels of COL3 and MMP9 (Figure 2H–J). In addition, by measuring the content of hydroxyproline and Masson staining in adipose tissue, it was found that overexpression of ITGA5 can significantly increase the content of collagen fibers in adipose tissue (Figure 2K,L). Based on the above research results, it showed that ITGA5 can promote adipose tissue fibrosis, and interference with the expression of ITGA5 can reduce adipose tissue fibrosis. Therefore, we speculated that leptin might alleviate adipose tissue fibrosis by inhibiting ITGA5.

### 2.3. ITGA5 Promotes Fibrosis in Adipocytes, Inhibition of ITGA5 Alleviates Fibrosis in Adipocytes

**Figure 3:**
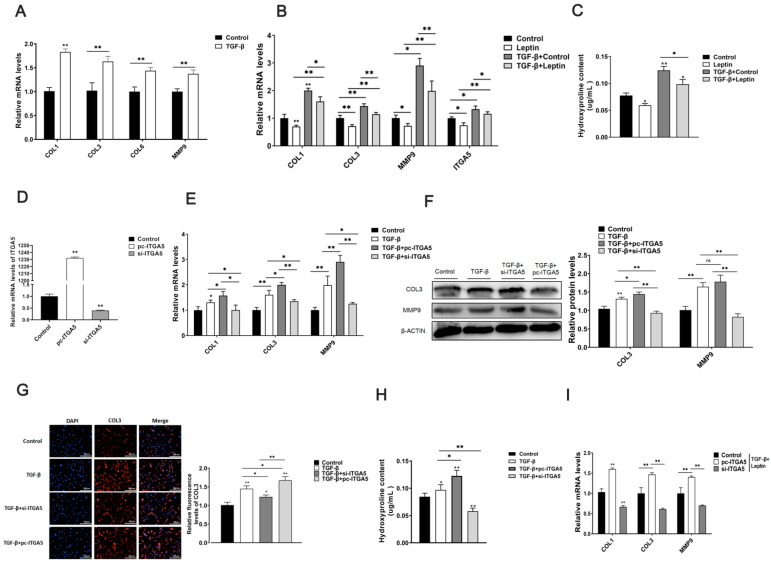
Leptin relieves fibrosis in adipocytes by inhibiting ITGA5. The cells were treated with TGF-β to construct a cell fibrosis model. Afterwards, it was treated with leptin. (**A**) The relative mRNA expression levels of COL1, COL3, COL6, MMP9, after TGF-β treated cells (*n* = 4). (**B**) The relative mRNA expression levels of fibrosis-related genes COL1, COL3, MMP9, and ITGA5 in adipocytes treated with TGF-β and leptin (*n* = 4). (**C**) The content of hydroxyproline after treating for TGF-β and leptin in adipocytes (*n* = 4). (**D**) The relative ITGA5 mRNA expression level after treatment with ITGA5 overexpression vector and interference fragments in adipocytes (*n* = 4). The cells were treated with TGF-β to construct a cell fibrosis model. Then the adipocytes were, respectively, treated with ITGA5 overexpression vector and interference fragments. (**E**,**F**) The relative expression levels of COL1, COL3, MMP9 mRNA, and COL3, MMP9 protein were detected (*n* = 4). (**G**) Immunofluorescence staining of COL3, scale bar: 200 μm (*n* = 4). (**H**) The content of hydroxyproline (*n* = 4). (**I**) The relative expression levels of COL1, COL3, MMP9 mRNA. Values are means ± SD, * *p* < 0.05, ** *p* < 0.01 compared with the control group.

To clarify that ITGA5 promoted adipose tissue fibrosis, we treated cells with 10 ng/mL TGF-β at the cellular level to construct a cell fibrosis model, and found that TGF-β can significantly promote the mRNA expression levels of adipocyte fibrosis-related genes (Figure 3A); this indicated that TGF-β could be used to build adipocyte fibrosis models. Further testing found that after TGF-β treatment, the mRNA expression levels of cell fibrosis-related genes COL1, COL3, MMP9, and ITGA5 increased significantly. Afterwards, it was treated with leptin; the mRNA expression of cell fibrosis-related genes and ITGA5 were significantly reduced (Figure 3B). At the same time, TGF-β treatment promoted the production of hydroxyproline in adipocytes. However, it was treated with leptin, and could reduce the hydroxyproline content of adipocytes (Figure 3C). These results indicate that TGF-β could be used to build adipocyte fibrosis models; afterwards, it was treated with leptin alleviated adipocyte fibrosis; meanwhile, ITGA5 was closely related to adipocyte fibrosis.

To further explore the effect of ITGA5 on adipocytes fibrosis, we constructed a fibrosis model by treating adipocytes with TGF-β, and then separately treated adipocytes with the ITGA5 overexpression vector and interference fragments. ITGA5 mRNA level detection revealed that ITGA5 overexpression and interference efficiency were extremely significant (Figure 3D). Further research results found that ITGA5 could promote the expression of COL1, COL3, and MMP9 mRNA and COL3, MMP9 protein (Figure 3E,F). The COL3 immunofluorescence test was consistent with the protein level test results (Figure 3G). Further testing found that ITGA5 can increase the content of hydroxyproline in adipocyte (Figure 3H). Furthermore, in the TGF-β-induced fibrosis model, after TGA5 interference and overexpression were treated with leptin at the same time, it was found that the therapeutic effect of the cells was better than that of leptin or iTGA5 alone in inhibiting fibrosis (Figure 3I). These results indicate that ITGA5 can promote adipocyte fibrosis, and leptin relieves fibrosis in adipocytes by inhibiting ITGA5.

### 2.4. ITGA5 Promotes Fibrosis in Adipocytes by Activating the PI3K-AKT Signaling Pathway

**Figure 4:**
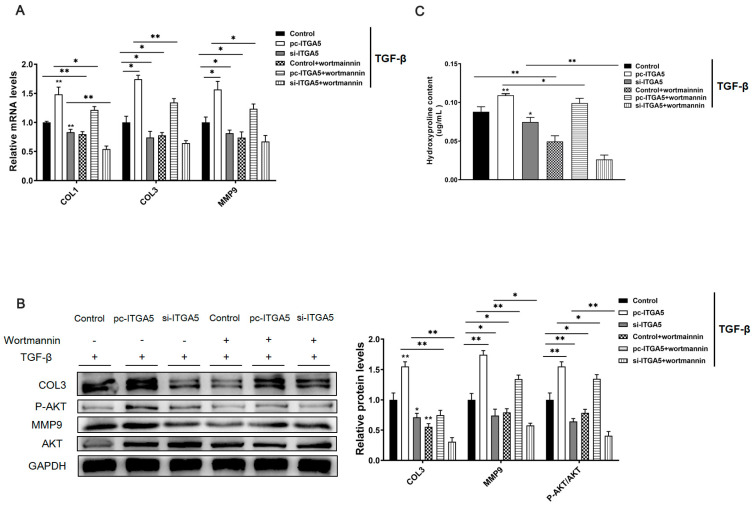
ITGA5 promotes adipocyte fibrosis by activating the PI3K-AKT signaling pathway. The adipocytes were treated with TGF-β to construct a cell fibrosis model. Then the adipocytes were transfected with ITGA5 overexpression vector and interference fragments, and the adipocytes were treated with wortmannin (an inhibitor of PI3K-AKT signaling pathway). (**A**) The relative mRNA expression levels of fibrosis-related genes COL1, COL3, and MMP9 (*n* = 4). (**B**) The relative protein expression levels of COL3, MMP9, p-AKT, and AKT (*n* = 4). (**C**) The content of hydroxyproline in adipocytes (*n* = 4). *n* = 4 in each group. Values are means ± SD, * *p* < 0.05, ** *p* < 0.01 compared with the control group.

According to KEGG analysis and existing research reports, our comprehensive analysis found that ITGA5 is closely related to the PI3K-AKT signaling pathway. To further determine the molecular mechanism of ITGA5 regulating fibrosis in adipocytes, we treated adipocytes with TGF-β to construct a cell fibrosis model. Then, adipocytes were transfected with ITGA5 overexpression vector and interference fragments, and treated with PI3K-AKT signaling pathway inhibitor wortmannin, detect the relative expression of fibrosis-related genes COL1, COL3, MMP9 in adipocytes, the content of hydroxyproline, and the phosphorylation level of AKT (p-AKT). The results showed that ITGA5 can significantly promote the mRNA and protein expression levels of fibrosis-related genes COL1, COL3, MMP9 in adipocytes, and the addition of wortmannin can significantly reduce this promotion trend (Figure 4A,B). Moreover, ITGA5 can significantly promote the phosphorylation level of AKT, and the addition of wortmannin can significantly inhibit this trend (Figure 4B). Further testing found that ITGA5 can significantly increase the content of hydroxyproline in adipocytes, but the addition of wortmannin can significantly reduce this promotion trend (Figure 4C). These findings indicate that ITGA5 promotes adipocyte fibrosis by activating the PI3K-AKT signaling pathway.

### 2.5. Leptin Promotes White Fat Browning and Relieves the Inhibitory Effect of TGF-β on Adipocyte Browning

**Figure 5:**
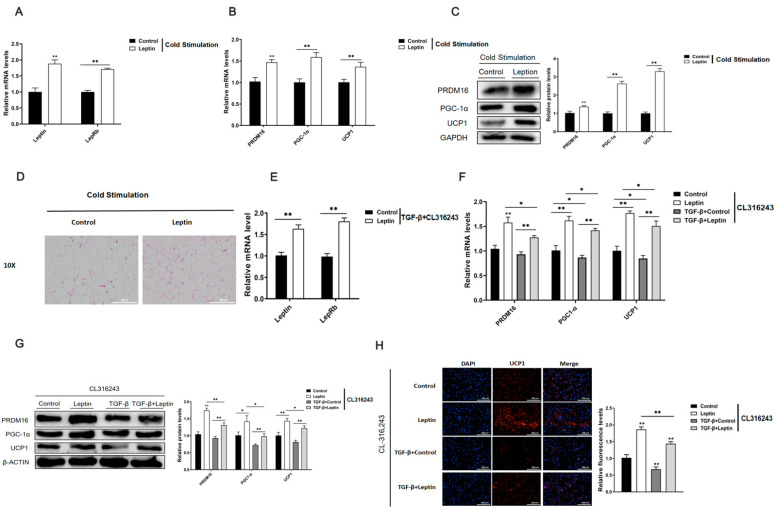
Leptin promotes browning of fat tissues and improves the inhibitory effect of TGF-β on browning of adipocyte. Eight-week-old C57BL/6J mice were injected with leptin for 7 d and exposed to a cold environment at 4 °C for 24 h. After the mice were sacrificed in the neck, the total RNA and protein of the inguinal adipose tissue were extracted. (**A**) The relative mRNA expression levels of leptin and its receptor (*n* = 4). (**B**,**C**) The relative mRNA and protein expression levels of adipose tissue browning-related genes PRDM16, PGC-1α, UCP1 (*n* = 4). (**D**) HE staining of adipose tissue section, scale bar: 200 μm (*n* = 4). We used TGF-β to build a cell fibrosis model, and, under the conditions, treated with leptin, and then treated with an adrenergic receptor agonist (CL316, 243) to induce browning of adipocytes. (**E**) The relative mRNA expression levels of leptin and its receptor (*n* = 4). (**F**,**G**) The relative mRNA and protein expression levels of adipocyte browning-related genes PRDM16, PGC-1α, UCP1 (*n* = 4). (**H**) Immunofluorescence staining of UCP1, scale bar: 200 μm (*n* = 4). Values are means ± SD, * *p* < 0.05, ** *p* < 0.01 compared with the control group.

Studies have found that adipose tissue fibrosis is closely related to white fat browning. To explore the effect of leptin on adipose tissue browning, firstly, eight-week-old C57BL/6J mice were injected with leptin for 7 d and exposed to a cold environment at 4 °C for 24 h. Afterward, the total RNA and protein of the inguinal adipose tissue were extracted. The results of the study found that leptin could significantly increase the mRNA relative expression levels of leptin and its receptors in adipose tissue (Figure 5A). Further testing found that leptin could significantly up-regulate the mRNA and protein expression levels of adipose tissue browning-related genes PRDM16, PGC-1α, and UCP1 (Figure 5B,C). Next, we combined with the results of HE staining of adipose tissue, it was found that leptin could significantly reduce the size of droplets in adipose tissue (Figure 5D). These results indicate that leptin promotes browning of white adipose tissue.

Furthermore, we explored the effect of leptin on adipocyte browning under fibrotic conditions. We first used TGF-β to construct adipocyte fibrosis model, afterwards, it was treated with leptin and an adrenergic receptor agonist (CL316, 243) to induce adipocyte browning. It was found that leptin up-regulated the relative mRNA expression levels of leptin and its receptor in adipocytes (Figure 5E). Further testing found that the browning-related genes PRDM16, PGC-1α, UCP1 mRNA and protein expression levels were significantly inhibited in the fibrosis model after TGF-β treatment. However, the opposite was true for treating with leptin. Further research found that leptinsignificantly reversing the expression of browning-related genes inhibited by TGF-β (Figure 5F,G). Immunofluorescence staining of UCP1 also supported these result (Figure 5H). The above test results show that leptin promotes white fat browning and relieves the inhibitory effect of TGF-β on adipocyte browning.

### 2.6. Down regulation of ITGA5 Alleviates the Inhibitory Effect of Browning Caused by Fibrosis

**Figure 6:**
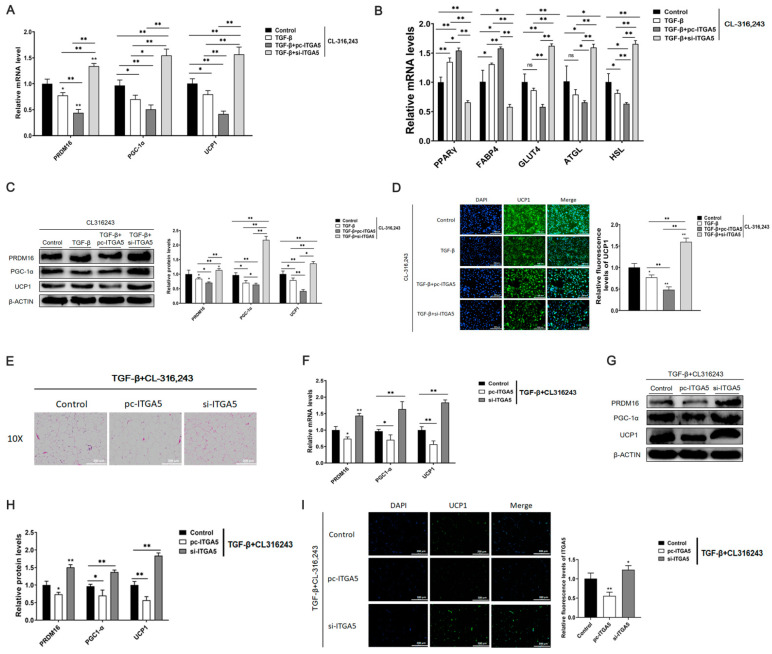
Down regulation of ITGA5 alleviates the inhibitory effect of browning caused by fibrosis. At the cellular level, a cell fibrosis model was constructed by TGF-β treatment, treated with CL316, 243, and then respectively treated with ITGA5 overexpression vector and interference fragment. (**A**,**B**) The relative mRNA expression levels of adipocyte browning-related genes PRDM16, PGC-1α, UCP1, PPARγ, FABP4, GLUT4, ATGL, and HSL (*n* = 4). (**C**) The relative protein expression levels of adipocyte browning-related genes PRDM16, PGC-1α, and UCP1 (*n* = 4). (**D**) Immunofluorescence staining of UCP1, scale bar: 200 μm (*n* = 4). At the in vivo level, the adipose tissue fibrosis model was constructed through HFD induced fat rats. Then the ITGA5 overexpression vector and interference fragment were injected subcutaneously for 7 days, and exposed at 4 °C for 24 h. We extracted the subcutaneous adipose tissue of the mouse groin. (**E**) HE staining of adipose tissue sections, scale bar: 100 μm (*n* = 4); (**F**–**H**) Relative mRNA and protein expression levels of adipocyte browning-related genes PRDM16, PGC-1α, and UCP1 (*n* = 4). (**G**) UCP1 immunofluorescence of adipose tissue sections, scale bar: 200 μm (*n* = 4). (**I**) Values are means ± SD, * *p* < 0.05, ** *p* < 0.01 compared with the control group.

To explore whether ITGA5 could affect white fat browning by affecting adipose tissue fibrosis, in vitro, a cell fibrosis model was constructed by TGF-β treatment; after treatment with CL316, 243, and used ITGA5 overexpression vector and interference fragment processing.We detected the mRNA and protein expression of browning-related genes, PRDM16, PGC-1α, and UCP1. The results showed that ITGA5 could significantly inhibit the mRNA and protein expression of browning genes, PRDM16, PGC-1α, and UCP1; moreover, the mRNA of lipid metabolism related genes PPARγ, FABP4, GLUT4, ATGL, and HSL also showed the same result (Figure 6A–C). Immunofluorescence staining of UCP1 further verified this result (Figure 6D).

At the in vivo level, mice were induced to construct the adipose tissue fibrosis model through a high-fat diet (HFD). Then the mice were injected with ITGA5 overexpression vector and interference fragment for 7 days, and then exposed at 4 °C for 24 h. Finally, the mouse inguinal adipose tissue was extracted. HE staining found that ITGA5 increases the size of lipid droplets in adipose tissue, while interference with ITGA5 could significantly reduce the size of lipid droplets (Figure 6E). Total RNA and protein were respectively extracted, and the effects of overexpression and interference with ITGA5 on the expression of browning genes were detected. The results showed that the interference of ITGA5 could significantly increase the relative mRNA and protein expression of browning-related genes PRDM16, PGC-1α, UCP1, while the overexpression of ITGA5 was the opposite (Figure 6F–H). Moreover, the immunofluorescence staining of adipose tissue UCP1 further confirmed this result (Figure 6I). These results indicate that ITGA5 significantly inhibited white fat browning in the fibrosis model; therefore, this also confirms our guess that leptin, through regulating the expression of ITGA5, alleviates browning caused by fibrosis.

## 3. Research Highlights

(1) Leptin relieves adipose fibrosis by inhibiting ITGA5;
(2) ITGA5 promotes adipocyte fibrosis by activating the PI3K-AKT signaling pathway, inhibiting ITGA5 could alleviate adipocyte fibrosis;
(3) Down regulation of ITGA5 alleviates the inhibitory effect of browning caused by fibrosis.

## 4. Discussion

Adipose tissue fibrosis is caused by obesity and other factors, which induce adipose tissue, to be in an environment of hypoxia and chronic inflammation for a long time, and eventually lead to the excessive deposition of ECM in adipose tissue, resulting in adipose tissue metabolism disorder.

In the process of adipose tissue fibrosis, adipose tissue ECM, HIF-1α, and inflammatory factors are in a state of continuous remodeling, leading to a significant increase in the expression of fibrosis-related genes such as COL1, COL3, COL6, and MMP9 in adipose tissue [31,32]. ECM in adipose tissue is mainly secreted by adipocytes, and macrophages play an important regulatory role in this process [33]. Therefore, when the expression of collagen fibers in adipocytes significantly increase, it may cause a large accumulation of ECM in adipose tissue and ultimately lead to adipose tissue fibrosis [34]. Studies have found that adipose tissue fibrosis usually occurs in obese mice fed with HFD and ob/ob mice lacking leptin gene; therefore, HFD and ob/ob mice are usually studied as a model of adipose tissue fibrosis. 

Leptin has been proven to play an important role in the fibrosis process of multiple organs; studies have found that leptin can reduce the expression of COL6 in human adipose tissue [35]. In ob/ob mice, adipose tissue is accompanied by inflammation and fibrosis, but injection of leptin can alleviate adipose tissue inflammation and reduce the expression of fibrosis-related genes [21,36]. Based on this, we speculate whether leptin can regulate adipose tissue fibrosis. Therefore, this experiment used leptin to treat mice, to explore the effect of leptin on the expression of adipose tissue fibrosis-related genes. Our study found that leptin treatment could significantly reduce the expression of fibrosis-related genes, which indicates that leptin can effectively alleviate the fibrosis of adipose tissue.

Integrin is a dimeric transmembrane protein consisting of an α subunit and a β subunit. Integrin α5 (ITGA5) is a member of the integrin family [37]. Studies have found that ITGA5, as the main receptor of fibronectin, can further activate downstream signaling pathways, such as PI3K-AKT and mitogen-activated protein kinase (MAPK) by binding downstream integrin-related kinases (ILK) [38]. Moreover, ITGA5 plays an important role in the synthesis of extracellular collagen. Studies have found that ITGA5 promotes the expression of collagen fibers in the extracellular matrix of the liver. After inhibiting ITGA5, it will reduce the expression of collagen in liver tissues, thereby alleviating the process of liver fibrosis [30]. ITGA5 can also interact with ECM as a receptor for fibronectin, and regulate the expression of ECM, such as collagen, by activating downstream signaling pathways [39]. In the liver, inhibiting the expression of ITGA5 can significantly alleviate liver fibrosis [40]. After treating adipocytes with leptin, we performed proteomic sequencing and found that the expression of ITGA5 was significantly reduced in the leptin treated group. Further research found that, in HFD mice and ob/ob mice, the expression level of ITGA5 also increased significantly along with the increase in the expression levels of COL1 and COL3, and other fibrotic genes. In addition, in the adipocyte fibrosis model constructed by TGF-β treatment, the expression level of ITGA5 significantly increased. Afterwards, it was treated with leptin; the expression level of ITGA5 significantly reduced. Furthermore, after treating adipocytes with ITGA5 overexpression vector and interference fragments, we found that overexpression of ITGA5 promoted the expression of fibrosis-related genes in adipocytes. In addition, we injected ITGA5 overexpression vector and interference fragments into mice, and detected the expression of adipose tissue fibrosis-related genes. The detection results were consistent with the detection results at the cellular level, further verifying the effect of ITGA5 on adipose tissue fibrosis. Finally, to further explore the molecular mechanism of ITGA5 affecting adipocyte fibrosis, we analyzed the sequencing results and the downstream signaling pathway mediated by ITGA5, and found that ITGA5 may be expressed through the PI3K-AKT pathway. To verify this conjecture, after treating adipocytes with the PI3K-AKT pathway inhibitor wortmannin, the adipocytes were further treated with the ITGA5 overexpression vector and interference fragments to detect the expression of fibrosis-related genes. The results show that when the PI3K-AKT pathway is inhibited, the effect of ITGA5 on the expression of fibrotic genes will be inhibited. Therefore, it is speculated that ITGA5 may promote the expression of adipocyte fibrosis-related genes by activating the PI3K-AKT pathway. Down-regulation of ITGA5 may reduce the activation of the PI3K-AKT pathway, reduce the expression of adipocyte fibrosis-related genes, and thereby alleviate adipose tissue fibrosis.

Leptin is a cytokine mainly secreted by adipocytes, and it plays an important role in lipid metabolism and tissue fibrosis [41,42]. Studies have found that when obesity, adipose tissue is often accompanied by chronic inflammation, and a large amount of adipose tissue ECM is remodeled, which further leads to adipose tissue fibrosis [19,32]. Adipose tissue fibrosis will further promote adipose tissue inflammation, leading to adipose tissue dysfunction. White fat browning is not only beneficial to fatty acid metabolism, but could also reduce adipose tissue inflammation caused by obesity [43,44]. At the same time, in the process of white fat browning, leptin not only stimulates the activation of sympathetic nerves by acting on the hypothalamus, further promoting the release of norepinephrine, and promoting the browning of white adipose tissue, but it also directly passed through its downstream signaling pathway, promoting the expression of PRDM16 and lipid deposition by inhibiting the Hh signaling pathway [6,45,46,47].

Current studies have found that the browning of white fat can effectively alleviate the fibrosis in adipose tissue. In addition, in brown adipose tissue, when inflammation and fibrosis occur, brown adipocytes will turn to white [48]. To study the effect of adipose tissue fibrosis on white fat browning, we used ITGA5 to explore the effect of leptin on fat browning. We treated mice with leptin, and found that leptin can significantly up-regulate the expression of browning-related genes. Further, in the cell fibrosis model constructed by TGF-β, the expression of browning-related genes was significantly inhibited, and leptin was able to reverse this effect. When using ITGA5 overexpression or interference fragments to treat adipocytes and mice, it was found that interference with ITGA5 would significantly promote the up-regulation of browning-related genes, while overexpression of ITGA5 will significantly inhibit the expression of browning-related genes. Overall, we speculate that leptin could promote adipose tissue browning by down-regulating the expression of ITGA5.

## 5. Materials and Methods

### 5.1. Animal Experiment

Eight-week-old C57BL/6J male mice were purchased from the Experimental Animal Center at the Fourth Military Medical University (Xi’an, China). Moreover, the use of the animals and mouse handling protocols were conducted following the guidelines and regulations approved by the Animal Ethics Committee of Northwest A&F University (Yangling, Shaanxi). Mice were housed at 2–5 per cage and provided ad libitum with water and a standard laboratory chow diet. Meanwhile, the animal room was maintained at a constant temperature of 25 ± 1 °C and humidity at 55 ± 5%, and 12 h light/12 h dark cycles.

### 5.2. Adipocyte Culture

The frozen 3T3-L1 adipose precursor cells (Shanghai Bio Leaf Biotech Co., Shanghai, China) were resuscitated, passaged, and plated. Take a 6-well plate as an example, Transfection is performed when the cell density reaches about 75%. About 6 h before transfection, we changed the medium to a serum-free medium without dual antibody. We added 2 μg of recombinant plasmid and 2 μL of transfection reagent to the anti-serum-free medium, vortex to mix, and let stand for 20 min. We added the mixed solution to a petri dish, put it in the incubator for 6 h, and then replaced it with normal culture medium. After 48 h, we added induction solution I (IBMX: 0.5 mmol/L, TOPSCIENCE, Shanghai, China; Dex: 1 μmol/L, Solarbio, Beijing, China; insulin: 10 μg/mL, TOPSCIENCE, Shanghai, China) for two days, then added induction solution II (insulin: 10 μg/mL) for 8 days. Every two days, we changed the induction liquid II once. After 8 days, the cells were collected for further processing.

### 5.3. Determination of Adipose Tissue Hydroxyproline

The hydroxyproline content of adipose tissue was determined using a hydroxyproline quantitative kit (Merck, Darmstadt, Germany). First, we weighed 30–100 mg of adipose tissue into a test tube, added 1 mL of hydrolysate, mixed well, covered, and boiled in a water bath for 20 min to hydrolyze the sample. Then, we adjusted the pH to about 7.0–7.8, and added distilled water to 10 mL. We took 3–4 mL diluted hydrolysate and added the appropriate amount of activated carbon, mixed well, centrifuged at 3500 rpm/min for 10 min, and carefully took 1 mL of supernatant for testing.

We took a blank tube, a standard tube, and a measuring tube, respectively, added 1 mL of double distilled water, standard application solution, test solution to each, and added 0.5 mL of Reagent I, mixed well, and let stand for 10 min. We added 0.5 mL of Reagent II, mixed well, and let stand for 5 min. We added 0.5 mL of Reagent III, mixed well, placed in a water bath at 60 °C for 15 min, centrifuged at 3500 rpm/min for 10 min after cooling, took the supernatant at a wavelength of 550 nm, 1 cm optical path, adjusted to zero with double distilled water, and measured the absorbance of each tube. The calculation formula is: hydroxyproline content (μg/mL) = $\frac{\mathrm{measured}\;\mathrm{OD}\;-\;\mathrm{blank}\;\mathrm{OD}}{\mathrm{standard}\;\mathrm{OD}\;-\;\mathrm{blank}\;\mathrm{OD}\;}\times \;\mathrm{standard}\;\mathrm{product}\;\mathrm{content}\;\left(5\;\mu g/\mathrm{mL}\right)\times \frac{\mathrm{total}\;\mathrm{volume}\;\mathrm{of}\;\mathrm{hydrolysate}\;\left(\;\mathrm{mL}\right)}{\mathrm{wet}\;\mathrm{tissue}\;\mathrm{weight}\;\left(\mathrm{mg}\right)}$.

### 5.4. Real-Time Quantitative PCR Analysis

Total RNA was extracted from eWAT or adipocytes with TRIpure Reagent kit (Takara, Dalian, China). Reverse transcription of 500 ng total RNA using M-MLV reverse transcription kit (Takara, Dalian, China). Moreover, primers were synthesized by Invitrogen (Invitrogen, Shanghai, China). Further quantitative PCR was performed in 25-μL reaction system containing specific primers and AceQ qPCR SYBR Green Master Mix (Vazyme, Nanjing, China). Amplification was performed in the ABI Step One Plus™ RT-PCR System (ABI, Carlsbad, CA, USA). The levels of mRNA were normalized in relevance to GAPDH. Moreover, the expression of genes was analyzed by method of 2^−ΔΔ*C*t^, ΔΔ = (CT_Target Gene_ − CT_GAPDH_) T − (CT_Target Gene_ − CT_GAPDH_) C. 

### 5.5. Western Blotting Analysis

Proteins (30 μg) were separated by SDS-PAGE, transferred to the PVDF nitrocellulose membrane (Millipore, Boston, MA, USA), blocked with 5% fat-free milk for 2 h at room temperature, and then incubated with primary antibodies in 5% milk overnight at 4 °C. COL3, MMP9 antibodies were all purchased from Wanlei (Wanlei, Shen Yang, China), and GAPDH from Bioworld (Bioworld, Nanjing, China). Rabbit HRP-conjugated secondary antibody (Baoshen, Beijing, China) was added and incubated at room temperature for 2 h. Then proteins were visualized using chemiluminescent peroxidase substrate (Millipore, Boston, MA, USA), and finally the blots were quantified using ChemiDoc XRS system (Bio-Rad, Hercules, CA, USA).

### 5.6. Immunofluorescence Assay

We treated the cells as required by the experiment, then fixed with 4% paraformaldehyde (MACKLIN, Shanghai, China). Next, we blocked with 5% BSA (WOLSEN, Shenzhen, China) in PBS for 1 h at room temperature. Then, the cells were incubated with rabbit polyclonal anti-COL3 primary antibody (Wanlei, Shenyang, China) at a dilution of 1:50 overnight, followed by incubation with fluorescein isothiocyanate-conjugated goat anti-rabbit IgG antibody (Boster, Wuhan, China) for 1 h at room temperature; we used DAPI for nuclear staining. Finally, we used a fluorescence microscope to observe and photograph the cells.

### 5.7. HE Staining

The paraffin sections were immersed in xylene (GHTECH, Guangdong, China), absolute ethanol (GHTECH, Guangdong, China), 90% ethanol, 80% ethanol, and 70% ethanol for dewaxing and rehydration. Afterward, we washed 3 times with 1×PBST, stain with hematoxylin (WOLSEN, Shenzhen, China). Then we immersed in 50%, 70%, and 80% ethanol, in sequence. After eosin staining (WOLSEN, Shenzhen, China), it was immersed in 80%, 90%, and absolute ethanol for gradient dehydration. After completion, we soaked in xylene (GHTECH, Guangdong, China) twice. Finally, we performed drying, mounting, and microscopy.

### 5.8. Immunohistochemistry

The dewaxing/rehydration operation of the slice was the same as the HE staining procedure. Then we immersed the slices in 1× antigen retrieval solution (LEAGENE, Anhui, China) for antigen retrieval. We placed the repaired sections into 3% H_2_O_2_ solution (Merck, Darmstadt, Germany) and incubated for 15 min in the dark. Then, we permeabilized with 0.1–0.2% Triton X-100 (Solarbio, Beijing, China) for 10 min. Next, we incubated for 1 h at room temperature with a blocking solution prepared with 5% goat serum (WOLSEN, Shenzhen, China). We discarded the blocking solution and incubated the primary antibody overnight at 4 °C. Next, we incubated the biotin-labeled secondary antibody (Boster, Wuhan, China) at 37 °C for 1 h. We incubated HRP-labeled streptavidin (Thermo Fisher, Shanghai, China) for 30 min at room temperature. We incubated DAB chromogenic solution (Solarbio, Beijing, China) at room temperature and dark for 5 min. We counterstained with hematoxylin (WOLSEN, Shenzhen, China) for 10 min, then differentiated with 1% hydrochloric acid (GHTECH, Shenzhen, China) and ethanol (GHTECH, Shenzhen, China) for 2 s. After using the weakly alkaline blue promoting liquid to reverse the blue, we immersed in 80%, 90%, and absolute ethanol (GHTECH, Shenzhen, China) for gradient dehydration. Finally, we immersed in xylene (GHTECH, Shenzhen, China), twice. Then, we dried, mounted, and performed microscopic inspection.

### 5.9. Masson Stain

The dewaxing/rehydration operation of the slice was the same as the HE staining procedure. We washed 3 times with distilled water, and then stained the nucleus with nuclear staining solution (WOLSEN, Shenzhen, China). After staining the nucleus, we used 1% hydrochloric acid alcohol (75% alcohol: concentrated hydrochloric acid = 99:1) (GHTECH, Shenzhen, China) to differentiate for 1 s, and the distilled water turned blue. After dyeing with the slurry dye solution (G.fan, Shanghai, China) for 30–60 s, the color separation solution (G.fan, Shanghai, China) was used for color separation, and then the counter dye solution (G.fan, Shanghai, China) was used for dyeing. Finally, dehydration and transparency, mounting and microscopy were performed.

### 5.10. Oil Red O Staining

Oil Red O staining was performed using Oil Red O kit (Solarbio, Beijing, China). Frozen sections were prepared, dried at room temperature for 15–20 min, incubated with 100% isopropanol for 5 min, and then incubated with 0.5% oil red O solution for 7–8 min; they were then washed with 85% isopropanol solution for 3 min. We dehydrated and washed; finally, we used mounting tablets to mount the slides and perform a microscopic examination.

### 5.11. Statistical Analysis

Statistical calculations were performed using SAS v8.0 (SAS Institute, Cary, NC, USA). Statistical significance was determined using the one-way ANOVA test. Comparisons among individual means were made by Fisher’s least significant difference (LSD) post hoc test after ANOVA. Data are presented as mean ± SD; *p*< 0.05 is considered significant.

## Data Availability

Exclude the declaration.

## Abbreviations

**Abbreviations**	**Name**
ITGA5	integrin alpha 5
TGF-β	transforming growth factor-β
PI3K	phosphatidylinositol 3 kinase
AKT/PKB	protein kinase B
UCP1	uncoupling protein 1
NPY	neuropeptide Y
PRDM16	PR domain-containing 16
JAK2	janus kinase 2
STAT3	signal transducer and activator of transcription
Hh	hedgehog
ECM	extracellular matrix
HFD	high-fat diets
DMEM	Dulbecco’s Modified Eagle Medium
GTF2IRD1	general transcription factor II-I repeat domain-containing protein 1
SDS-PAGE	sodium dodecyl sulfate-polyacrylamide gel electrophoresis
PVDF	polyvinylidene fluoride
COL1	collagen i
COL3	collagen iii
COL6	collagen vi
MMP9	matrix metalloproteinase 9
PGC-1α	proliferator-activated receptor-gamma coactivator-1-alpha
IBMX	3-isobutyl-1-methylxanthine
MAPK	mitogen-activated protein kinase
DEX	dexamethasone
